# With Age Comes Maturity: Biochemical and Structural Transformation of a Human Centriole in the Making

**DOI:** 10.3390/cells9061429

**Published:** 2020-06-09

**Authors:** Catherine Sullenberger, Alejandra Vasquez-Limeta, Dong Kong, Jadranka Loncarek

**Affiliations:** Laboratory of Protein Dynamics and Signaling, NIH/NCI/CCR, Frederick, MD 21702, USA; alejandra.vasquezlimeta@nih.gov (A.V.-L.); dong.kong@nih.gov (D.K.)

**Keywords:** centrosome, centriole duplication, centriole elongation, centriole maturation

## Abstract

Centrioles are microtubule-based cellular structures present in most human cells that build centrosomes and cilia. Proliferating cells have only two centrosomes and this number is stringently maintained through the temporally and spatially controlled processes of centriole assembly and segregation. The assembly of new centrioles begins in early S phase and ends in the third G1 phase from their initiation. This lengthy process of centriole assembly from their initiation to their maturation is characterized by numerous structural and still poorly understood biochemical changes, which occur in synchrony with the progression of cells through three consecutive cell cycles. As a result, proliferating cells contain three structurally, biochemically, and functionally distinct types of centrioles: procentrioles, daughter centrioles, and mother centrioles. This age difference is critical for proper centrosome and cilia function. Here we discuss the centriole assembly process as it occurs in somatic cycling human cells with a focus on the structural, biochemical, and functional characteristics of centrioles of different ages.

## 1. Introduction

**Figure 1:**
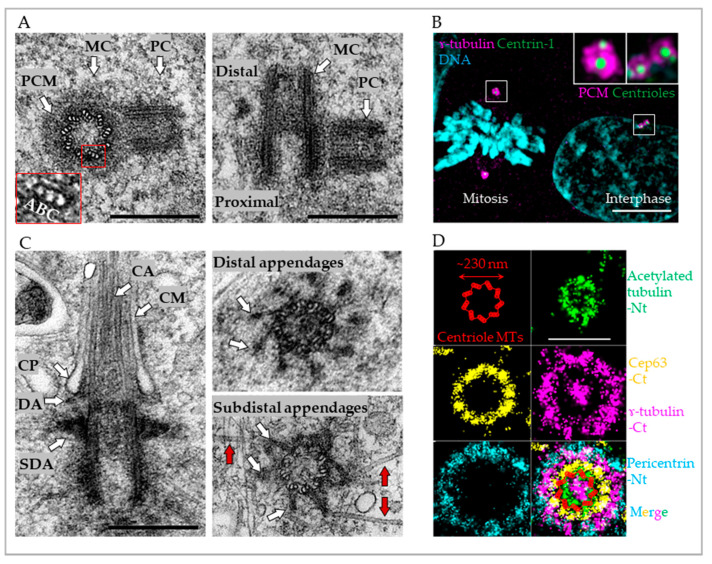
The organization of mature centrioles and centrosomes. (**A**) Left: Electron micrograph of a mother centriole (MC, in cross-section) associated with a procentriole (PC, in longitudinal section) from an mIMCD3 (mouse inner medullary collecting duct) cell. Pericentriolar material (PCM) is visible around the MC. One microtubule (MT) triplet built of A, B, and C MTs is enlarged in the red box. Right: Electron micrograph of a mother centriole (MC) and associated procentriole (PC) from an mIMCD3 cell. Both centrioles are sectioned longitudinally. (**B**) Centrosomes in mitotic or interphase human cycling retinal pigmental epithelial (RPE-1) cells. Two mitotic centrosomes have expanded pericentriolar material (PCM), here labeled by anti-γ-tubulin antibody, and are positioned on the opposite poles of the mitotic spindle. Each centrosome contains two centrioles, labeled with Centrin1-GFP. Chromosomes are aligning in the middle of the cell. In the interphase cell, two centrosomes, each harboring a duplicated mother centriole, are positioned near the nucleus and are associated with less abundant PCM. (**C**) Electron micrograph of a mature mother centriole with distal appendages (DA), subdistal appendages (SDA), and a cilium from a serum-starved RPE-1 cell. Ciliary axoneme (CA) is extending from the centriole distal end. DAs are adjacent to a ciliary pocket (CP), which continues into the ciliary membrane (CM) and surrounds the CA. Right: Cross sections through the region of centriole containing DAs (top) or SDAs (bottom) associated with MTs (red arrows). Electron microscopy was performed as described in Ref. [18]. (**D**) A montage of Stochastic Optical Reconstruction Microscopy (STORM) images of four centrosomal proteins immunolabeled as indicated and superimposed, to illustrate toroidal organization of centrosomal components with respect to centriole MTs (red). STORM and conventional microscopy were performed as described in Ref. [19] and using the antibodies as follows: γ-tubulin: Sigma; T5326, Cep63: Millipore; 06–1292, Pericentrin: Abcam; ab4448, and acetylated tubulin: Sigma; T7451. Scale bars: 400 nm in (**A** and **C**); 5000 nm in (**B**); 400 nm in (**D**).

The centrosome is a major microtubule (MT)-nucleating center of vertebrate cells and functions in controlling cell and tissue architecture, cell signaling, cell proliferation, and cell motility [1,2,3,4,5,6]. Each of the two centrosomes present in a cell is comprised of a core component, a centriole, which is a MT-based cylindrical structure, and a surrounding proteinaceous matrix called pericentriolar material (PCM) that is organized by the centriole (Figure 1A). During cell division, two centrosomes organize the two poles of the mitotic spindle, a transient MT-based structure that mediates chromosome segregation (Figure 1B). Because of their essential role in segregation of genetic material to two sister cells during mitosis, it is vital that cycling cells contain exactly two centrosomes. In quiescent cells, the older of the two centrioles can convert into a basal body and assemble a primary cilium on the cell surface (Figure 1C), which is an antenna-like organelle that senses and transmits extracellular signals, linking them with intracellular signaling pathways [7,8,9,10]. Numerical or structural aberrations of centrioles can initiate tumorigenesis and are present in most cancers. Additionally, mutations in a large set of centrosome-associated proteins are linked to a group of hereditary diseases commonly known as ciliopathies (reviewed in [5,11,12]).

Centrosomes are highly ordered organelles with a complex assembly process. In this review, we illustrate major steps of centriole and centrosome assembly as they occur in cycling human cells, with emphasis on the gradual metamorphosis of a centriole from a small function-less procentriole to a fully mature centriole capable of organizing a functional centrosome and a cilium. Throughout the article, we mostly refer to findings pertaining to human centrioles. However, due to evolutionary conservation of centriole structure and its assembly processes [13,14,15,16,17], we sometimes refer to the findings obtained from other organisms. We could not reference all publications that explore centriole assembly and structure and we apologize to those whose work will not be explicitly cited. Additionally, this article does not discuss many interesting aspects of centrosome biology. Thus, we direct the readership toward other publications centering on these herein undiscussed topics.

## 2. The Organization of Mature Centrioles and Centrosomes

In this chapter, we will provide a brief description of mature centrioles and centrosomes of mammalian cycling cells. Typical mammalian centrioles are ~230 nm-wide and ~500-nm long cylinders. Their walls are built of nine sets of microtubule (MT) triplets made from one full MT (tubule A) and two partial MTs (tubules B and C), organized with a precise nine-fold symmetry (Figure 1A) [16,20,21]. Centrioles are polarized along their longitudinal axis. The PCM localized to the proximal end mediates centrosomal functions such as MT nucleation and the assembly of new centrioles (Figure 1A) [22,23]. Microscopy-based mapping of individual protein domains in sub-resolutional scale has revealed a fascinating order in the organization of PCM components. Using antibodies targeting different epitopes of the same protein or N-terminally and C-terminally fused fluorescent tags, microscopy reveals that numerous PCM components are arranged in an extended conformation with one part of the molecule closer to the centriole and the other extending into the PCM. Many PCM components often associate with both centriole MTs and other PCM components resulting in the distribution of PCM proteins in circles of discrete diameters surrounding the centriole (Figure 1D) [24,25,26,27,28,29].

On their distal end, fully mature centrioles carry two types of electron dense projections called distal and subdistal appendages (DAs and SDAs, respectively, Figure 1C). Distal appendages reflect the nine-fold organization of centrioles and they appear as nine ~130 nm-long electron dense protrusions forming ~100 nm from the distal centriole end [19,20,21]. During ciliation, DAs are required for the attachment of ciliary vesicles to centrioles and their subsequent fusion with the cytoplasmic membrane and are thus, essential for ciliogenesis [30,31,32,33,34]. Subdistal appendages, which are formed just below DAs, are usually robust electron densities that are highly variable in shape and in number (Figure 1C) [35,36]. On their periphery, SDAs are associated with MTs, which regulate MT centrosome positioning [37,38,39,40,41] and cohesion [42].

The exact number of centrosomal proteins is not known and varies between different cell types. While novel centrosomal proteins are still being discovered, about 200 core centrosome proteins (systematized in a centrosome data base, [43,44]) have been identified through proteomic analyses [45,46,47,48]. The stoichiometry and intra-centrosomal localization of centrosomal components changes during the cell cycle and is dependent upon regulated transcription, degradation, and posttranslational modifications [49,50,51,52,53].

## 3. Assembly and Maturation of a Centriole Requires Three Consecutive Cell Cycles—A Brief Overview

**Figure 2:**
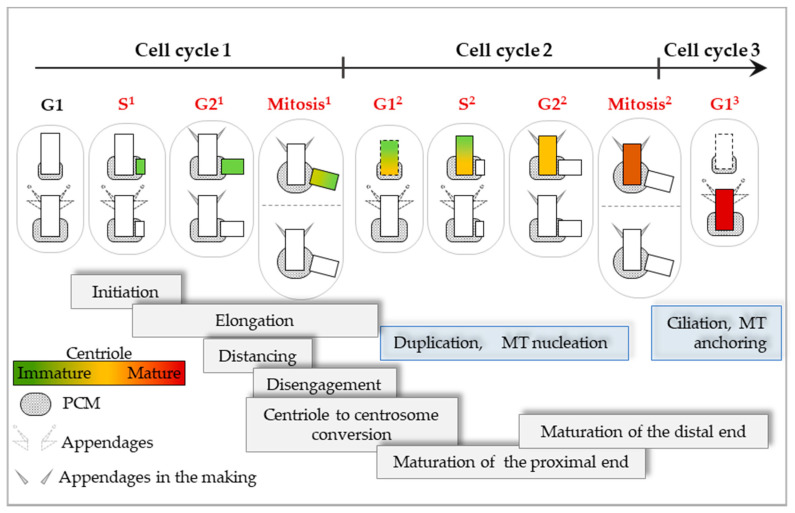
Assembly and maturation of a human centriole in cycling cells requires three consecutive cell cycles. After mitosis, cells inherit one partially mature and one fully mature centriole (with appendages). Both centrioles duplicate at the beginning of S phase. Scheme emphasizes one centriole from its initiation (green) in S phase of cell cycle 1 (S^1^) through its elongation and progressive maturation, until its final maturation in G1 of cell cycle 3 (G1^3^) (dark red with appendages). Gray boxes indicate the major assembly and maturation events that occur in each cell cycle. New functions acquired by the centriole at different stages of maturation are indicated by blue boxes, including duplication (cell cycle 2), MT nucleation (cell cycle 2), ciliation (cell cycle 3) and MT anchoring (cell cycle 3).

**Figure 3:**
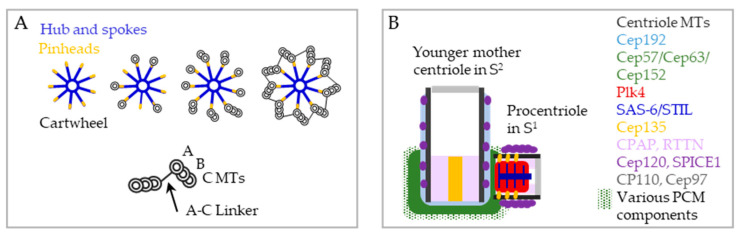
Procentriole initiation and MT assembly. (**A**) The first structure in the assembly of a procentriole is a nine-fold symmetrical cartwheel (top left). The three most distinguishable features of the cartwheel are the central hub, nine spokes, and nine pinheads that connect the cartwheel with microtubules. A, B, and C microtubules (MTs) are gradually assembled around the cartwheel. MT triplets are presumably stabilized by an A-C linker formed between the A and C MTs of adjacent MT triplets. The biochemical nature of the linker is unknown. (**B**) Localization of proteins involved in procentriole initiation and elongation on a mother centriole in S^2^ and a procentriole in S^1^. Mother centriole associated Cep192, Cep63, and Cep152 recruit Plk4 to the centrosome. After Plk4 associates with STIL, a Plk4/STIL focus assembles near the mother centriole and recruits cartwheel protein SAS-6 and Cep135. CPAP, Cep120, and SPICE1 promote centriole elongation while CP110 and Cep97, localized at the centriolar cap, prevent over-elongation.

**Figure 4:**
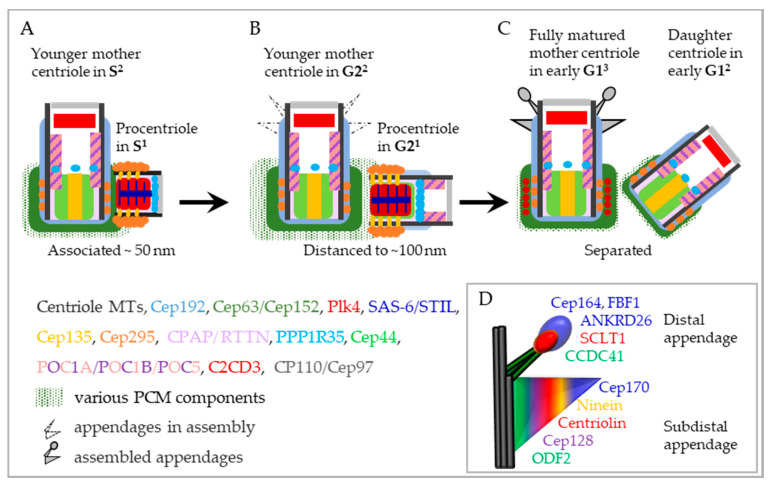
Localization of proteins involved in procentriole maturation from S^1^ to G1^2^. (**A**) In S^1^, the procentriole is adjacent to a mother centriole and lacks PCM. On their proximal end, in addition to the cartwheel, several proteins such Cep135, CEP295, CPAP, RTTN, and PPP1R35 are important for future procentriole stabilization. The younger mother centriole, in S^2^, is associated with PCM components and lower levels of Cep295 and PPP1R35. In addition, more distally, it contains POC1A/B, POC5, and C2CD3, which are important for the assembly and organization of triplet microtubules. (**B**) In G2^1^, procentrioles assemble their distal end, marked by the recruitment of new luminal proteins such as Cep44, POC1A/B, POC5, and C2CD3, which contribute to the structural integrity of the centriole. Additionally, Cep192 is recruited to the outer MT wall of the procentriole. The procentriole and the mother centriole are at a larger distance away from each other. In G2^2^, the younger mother centriole accumulates more PCM components at its proximal end, and on its distal end, starts accumulating inner subdistal and distal appendage components. (**C**) In G1^2^, procentrioles, which lose their cartwheel in mitosis, stabilize by recruiting various PCM components. In G1^3^, the younger mother centriole completes its maturation cycle by recruiting outer subdistal and distal appendages. (**D**) Localization of appendage proteins on fully mature mother centriole in G1^3^.

The centriole assembly process is unique amongst organelles in the sense that it requires three consecutive cell cycles to be completed. Once formed, centrioles persist through many generations of cycling cells [54]. The assembly of new centrioles (herein referred to as procentrioles) begins around the time of S phase entry in a process called centriole duplication (Figure 2 and Figure 3). The exact timing of procentriole initiation is still debatable since some studies argue that it occurs in late G1 phase [55] and others in early S phase [20,56]. While this conundrum still has to be resolved, in this review, we will adhere to the scenario that procentriole initiation occurs in early S phase. During duplication, one new procentriole initiates orthogonally near the proximal region of each of the two resident centrioles (herein referred to as mother centrioles). After their initiation, procentrioles elongate during S^1^, G2^1^, and mitosis^1^, and change their biochemical composition. They remain in the same centrosomal complex with mother centrioles until the end of mitosis^1^, after which they physically separate (disengage [57]) and organize their own PCM components, establishing a centrosome. We will refer to such disengaged procentrioles as daughter centrioles. From G1^2^ until the ensuing S phase (S^2^), the daughter centriole accumulates PCM components including those required for the initiation of their own procentriole. As a result, both the older mother centriole and the daughter centriole (from this point herein referred to as a younger mother centriole) duplicate upon S^2^ phase entry (Figure 2 and Figure 4). In the ensuing S^2^ and G2^2^ phases, the younger mother centriole accumulates additional PCM allowing it to enter mitosis with levels of PCM comparable to the older mother centriole (Figure 2 and Figure 4). In parallel to the maturation of their proximal end, in G2^2^ phase, the distal end of a younger mother centriole begins to associate with a set of proteins that will result in the structuring of subdistal and distal appendages, which are responsible for MT anchoring and ciliation, respectively (Figure 2 and Figure 4). Structuring of centriole appendages continues until G1^3^, and it represents the last step in the assembly of a centriole which was initiated two cell cycles ago. Only at this point has the centriole, and the centrosome which it organizes, gained a full range of functions.

By staggering centriole maturation over three consecutive cell cycles and gradually adding specific functions to them, cells ensure that two centrioles/centrosomes are capable of duplication and spindle formation, but only one centriole, the oldest, can ciliate. Such an orderly assembly process is unique amongst organelles and is a result of a remarkable coordination between the cell cycle and the centriole cycle machinery [58,59].

## 4. Morphological and Biochemical Changes of Centrioles during Assembly and Maturation

In this chapter, we will discuss the milestones, as well as the ultrastructural and biochemical changes, that occur on a typical centriole in a cycling somatic human cell, from its procentriole stage until its final transformation into a fully mature centriole.

### 4.1. From Procentrioles to Daughter Centrioles

#### 4.1.1. Procentriole Initiation—Ultrastructural Aspect

By electron microscopy, newly formed procentrioles can be detected ~50 nm from the proximal end of mother centriole wall. They appear as electron dense ~190 nm-wide circular assemblies that contain a centrally positioned nine-fold-symmetrical cartwheel and surrounding electron dense material, where nine sets of MT singlets, doublets, or triplets can be observed, depending on the stage of their development (Figure 3A). The procentriole cartwheel (reviewed in [60,61]) has three clearly distinguishable ultrastructural elements: a ~22 nm-wide central hub, nine spokes which radially emanate from the hub, and nine pinheads which connect the distal ends of the spokes with the procentriole’s innermost tubule, the A tubule (Figure 3A) [20,21,62].

A major component of the cartwheel is the protein SAS-6, which oligomerizes to generate a hub and nine spokes that emanate out of it. It is likely that another component of the spokes and the pinhead structure is Cep135 (Bld10p in *Chlamydomonas* and Bld10 in *Drosophila* [63,64,65,66,67]). The cartwheel provides stability and nine-fold symmetry to centrioles and is considered to be a scaffold for the formation of procentriole MTs. Both SAS-6 and Cep135 are necessary for procentriole assembly [68,69]. However, in human and *Chlamydomonas* cells carrying various SAS-6 and Cep135 mutations, cartwheel symmetry is perturbed, yet centriole MTs can still form as do procentrioles, albeit with a lower precision [66,70,71]. The cartwheel elongates during the cell cycle and by the end of G2^1^ phase it occupies ~180 nm of a procentriole’s ~280 nm length [72].

Ultrastructural studies of mammalian procentrioles show that MT triplets form progressively: the first to form is the A tubule, followed by the B, and C tubules. Cryo-electron tomography of procentrioles isolated from human lymphoblastoid cells [73] suggests that the A tubule elongates in a proximal-distal direction and serves as a template for the assembly of the B tubule, the elongation of which can be bidirectional. Similarly, the B tubule then templates bidirectional assembly of the C tubule. The formation of each MT triplet in human procentrioles is suggested to be independent [73] contrary to the synchronous buildup of all nine MT blades seen in some other species [74]. Additional analysis would be needed to understand whether this scenario of tubule incorporation universally applies to procentrioles in all human cell types. The literature offers ample descriptions of human procentrioles at stages containing MTs, but early stage procentrioles without MTs are poorly documented by electron microscopy, indicating that MT incorporation occurs relatively rapidly after procentriole initiation. However, a precise timing of A, B, and C tubule incorporation during the cell cycle still needs to be unraveled.

Once procentriole MT triplets are assembled, the inner A tubule and the outer C tubule from the adjacent MT triplets are connected by a linker (Figure 3A), which remains detectable on the proximal end of the centriole thereafter [20,21,62,75,76,77]. The composition and the exact function of the A-C linker are not known. POC1 has been proposed as a potential linker protein since, in *Tetrahymena*, its depletion slightly perturbed inter-triplet linkage and the integrity of basal bodies [78]. In addition to the more proximal A-C linker, there are additional electron densities that connect adjacent A tubules on the middle and distal parts of centrioles [20,79].

Until mitosis^1^, the angle between a MT triplet and the procentriole radius passing through the A tubule is smaller (~30°) than that of mature centrioles (~45°). In vertebrates, the cartwheel disappears from procentrioles in mitosis^1^, which is accompanied by a slight widening of the procentriole lumen and a change in the radial angle of procentriole MT triplets, such that by the end of mitosis^1^, a procentriole’s diameter is similar to the diameter of mature centrioles [20,80].

#### 4.1.2. Procentriole Initiation: Regulation and Required Proteins

Cartwheel assembly, which marks procentriole initiation, is triggered by the interaction of Polo-like kinase 4 (Plk4 [81], SAK in *Drosophila* [82], and Zyg-1 in *C. elegans* [83]) and SCL-interrupting locus protein (STIL [84,85], Ana-2 in *Drosophila* [86,87,88], and Sas-5 in *C. elegans* [89]), a procentriole initiator whose levels rise in the cytoplasm of cells approaching S phase [90,91]. In proliferating cells, Plk4, which is always present in the cytoplasm, regulates both its activity and its levels through trans-autophosphorylation of its kinase domain and autophosphorylation of its degron [92,93,94,95,96,97,98]. Its centrosomal localization is mediated by PCM components Cep192, Cep57, Cep63, and Cep152, which are localized around the proximal ends of mother centrioles (Figure 3B) [27,99,100,101,102]. On unduplicated mother centrioles in G1 phase, Plk4 is distributed around the proximal end of centrioles [103]. As cells approach S phase, Plk4 and STIL begin to associate. Phosphorylation of STIL by Plk4 further promotes their association and, in addition, protects Plk4 from degradation, resulting in an increase in the local Plk4/STIL concentration [104,105,106,107,108] and a change in Plk4 localization from a ring around the mother centriole to a single focus [103]. Once a PLK4/STIL focus is established in the vicinity of the mother centriole, the formation of other foci is inhibited by a still poorly understood molecular mechanism (for discussions see [85,109,110,111,112]). Phosphorylated STIL additionally recruits SAS-6 to the Plk4/STIL focus [105,106,113]. This promotes the self-oligomerization of SAS-6 into a nine-fold cartwheel scaffold that demonstrates lateral stacking and associates with other centriolar components (Figure 3) [70,114,115]. Downstream from Plk4/STIL/SAS-6-driven cartwheel assembly, the incorporation of other proteins such as Centrosomal-P4.1-associated-protein CPAP (also known as CENPJ and SAS-4 in *Drosophila* and *C. elegans*), is needed to start the assembly of procentriole MTs. Interestingly, it was recently shown that phosphorylation of STIL by Plk4 also plays an important role in the recruitment and stabilization of CPAP [116].

#### 4.1.3. Proteins Involved in Procentriole Elongation

Like cytoplasmic MTs, centriole MTs are also built of α- and β-tubulins. However, while cytoplasmic MTs are highly dynamic, centriole MTs are stable and once formed do not exchange tubulin with the cytoplasmic pool [117,118]. In mammalian cultured cycling cells, most procentriole MT growth occurs from early S^1^ until the end of mitosis^1^ [20,72,117,119,120]. Available studies disagree about whether mammalian procentrioles grow to their final length before or after G1^2^, although this disagreement may reflect cell type-specific differences in the dynamics of procentriole elongation or variability in experimental approaches used to assess centriole length. For instance, in human HeLa cells, procentrioles reach ~75% of their future length by mitosis^1^, and elongate to ~85–90% of their final length by the end of mitosis^1^ [72], similar to human lymphoblastic cells KE37 cells [119]. Centrioles of porcine kidney epithelial LLC-PK cells, on the other hand, do not incorporate biotinylated tubulin after G1^2^ [117].

It is still unknown what imparts this slow growth and stability to procentriole MTs or how centriole elongation is regulated. One major difficulty in studying procentriole elongation is that during procentriole assembly, elongation of MTs occurs in parallel with their stabilization and the procentriole’s biochemical maturation. In addition, the same proteins can be involved in several parallel processes during procentriole assembly. For instance, CPAP is needed for procentriole initiation, MT elongation, and PCM tethering to centrioles [91,121,122,123,124,125,126]. So, it is often impossible to perturb only one aspect of early procentriole assembly without affecting another.

In terms of its role in MT elongation, one of the best characterized proteins is CPAP. In humans, CPAP localizes in the lumen of procentrioles and in the lumen and PCM of mother centrioles (Figure 3B) [24,68]. CPAP associates with multiple procentriole-associated proteins such as Cep120, SPICE, Cep135, and Centrobin, which all contain MT-binding domains and positively regulate procentriole elongation [121,122,123,127,128,129,130]. Depletion of either CPAP, Cep120, SPICE1, or Cep135, or overexpression of CPAP or Cep120, results in shorter or elongated centrioles, respectively. On its N terminus, CPAP contains a MT binding domain (MBD) and an α/β-tubulin binding domain (PN2–3), which are both required for MT elongation [123,131,132,133]. The C-terminal portion of PN2–3 is suggested to act as a molecular lid which restricts MT growth [131,132]. However, how the PN2–3 region is regulated in vivo and how CPAP operates in the context of other centriolar proteins required for procentriole elongation is not clear.

Two proteins that ‘cap’ distal centriole ends, CP110 and Cep97, counteract elongation of centriole MTs and their depletion results in over-elongation of centriole MTs, mimicking the effects of CPAP overexpression [122,134,135]. Over-elongation of older centrioles is also somehow counteracted by a component of the distal centriole end, Ofd1 [136]. Finally, a group of centriolar proteins including RTTN [137] (Ana3 in *Drosophila* [138]), PPP1R35 [139,140], Cep295 [141], POC5 [142], and C2CD3 [143,144], which are situated in the vicinity of procentriole MT walls, are all critical for the formation of full-length centrioles. RTTN, Cep295, and PPP1R35 are incorporated at the proximal ends of procentrioles in S^1^ phase, while POC5 and C2CD3 are incorporated in G2^1^ phase and are more distal (Figure 4). It is possible that at least some of these proteins contribute to centriole elongation by promoting their overall structural integrity and maturation rather than being directly involved in MT nucleation (discussed in Chapter 5).

#### 4.1.4. Regulation of Procentriole Elongation

Centriole length is considered to be relatively constant for a particular cell type, although it may drastically vary between cell types of the same organism and between species. Centrioles of somatic human cells are ~500 nm-long, but it is not understood how centrioles achieve this target size. In *Drosophila* embryos, where the cartwheel occupies the entire length of ~200 nm-long centrioles, the rate and the period of procentriole elongation is regulated by Plk4, the same kinase that is responsible for cartwheel initiation [145]. However, human centrioles are longer, and their cartwheel occupies only the proximal procentriole end, meaning that their distal end, which elongates in G2^1^ phase and mitosis^1^, elongates beyond the cartwheel and in association with a different set of luminal and MT-associated proteins (Figure 4). Recent analysis of a series of human cell lines showed that in the same cell population, and sometimes within the same cell, mature and functional centrioles can range in their length from ~330 to ~600 nm [72]. Further, arresting cells in G2 phase and mitosis, two cell cycle phases that normally support faster procentriole elongation [72], resulted in a stochastic over-elongation of procentrioles. The extent of over-elongation was variable between cells of the same population and between different cell types. This implies that human cycling cells do not have an active monitoring system for centriole length. Instead, the rate of centriole growth could depend on the local stoichiometry of elongation factors and the time a centriole spends in a specific phase of the cell cycle.

Two members of the Polo-like kinase family, Plk1 and Plk2, have been shown to positively regulate procentriole elongation in human cells [72,146]. In cycling human cells, the activity of Plk1 is low in G1 and early S phases, increases in late S and G2 phases, and peaks around the time of mitotic entry [147]. Plk1 is mostly known for its mitotic function and mitotic maturation of centrosomes [148,149,150,151]. However, Plk1 inhibition from early S^1^ phase yielded procentrioles with shorter cartwheels and shorter MTs in mitosis and prevented procentriole over-elongation during mitotic arrest. Over-expression of Plk1 in cycling or S phase arrested human cells yielded longer than average centrioles with longer distal and/or proximal ends and displaced appendages [72]. In cycling cells, the peak of Plk1 activity also coincides with the faster phase of procentriole elongation. So, it is plausible that in human cells, Plk1, similarly to Plk4 in *Drosophila* [145], influences the rate of centriole elongation. Plk1 inhibition could reduce but not prevent procentriole elongation, suggesting that in human cells multiple regulators participate in centriole elongation. It is yet to be understood how Plk1 and Plk2 influence centriole elongation, but it is noteworthy that both kinases can phosphorylate CPAP [146,152]. Plk2, which is mostly active in early S phase [153], promotes procentriole elongation through phosphorylation of CPAP S589 and S595 [146], although further molecular mechanisms are unknown.

#### 4.1.5. Mother Centriole–Procentriole Distancing and Separation

Procentrioles form in the vicinity of the proximal end of mother centrioles. Under physiological conditions, procentrioles form ~50 nm from the mother centriole MT wall (Figure 1A and Figure 4A). When the procentriole is closely associated with the mother centriole, the two are said to be engaged. Maintaining a close association between a mother centriole and a procentriole during interphase is a critical factor in regulating centriole number, since the presence of the procentriole inhibits mother centriole reduplication. This centrosome intrinsic block to mother centriole reduplication was originally proposed by Wong and Stearns [154], who showed, performing a series of cell fusion experiments, that only mother centrioles which were not associated with procentrioles could duplicate in a duplication-permissive environment. Ablations of procentrioles from mother centriole–daughter complexes using a laser microbeam further established that inhibition of mother centriole reduplication originates from the presence of a procentriole in its vicinity [155]. Additionally, a series of chemical and genetic manipulations of Plk4 and STIL established that phosphorylation of STIL by Plk4 is required for its maintenance at the procentriole [106] and for the sustained reduplication block [156].

As the cell cycle progresses, the physical distance between the procentriole and the mother centriole increases and by the end of G2^1^ (prophase), the distance between the centrioles can reach >100 nm (Figure 4B) [20,157]. At this stage, the proximal ends of procentrioles are still embedded in the mother centriole PCM, which begins to expand in preparation for mitosis. It is not clear whether such distancing occurs gradually or abruptly in G2, but it has been shown that it requires Plk1 [157]. The importance of a tight mother centriole–procentriole association during interphase becomes obvious in the context of pathological reduplication of centrioles, which is also promoted by Plk1. Analysis of reduplicating centrioles by time-lapse microscopy [155] and correlative time-lapse electron microscopy [157] revealed that premature distancing of procentrioles to ~80–90 nm precedes, and is necessary, for centriole reduplication. It is not clear how Plk1 drives mother centriole–procentriole distancing during interphase, but one hypothesis is that Plk1 promotes unscheduled procentriole maturation program (regulation of procentriole maturation by Plk1 is discussed in Chapter 5), leading to their premature ousting from the mother centriole PCM due to spatial limitations created by the growing PCM of the maturing procentriole [157]. However, this hypothesis needs to be experimentally corroborated.

After mitotic entry until anaphase, two distanced centrioles still move as a part of the same centrosome, although their association within the centrosome is no longer stringent [157], suggesting that the two centrioles are only loosely held together by the expanded PCM. Indeed, in cells depleted of the PCM component Pericentrin, centrioles tend to prematurely separate during mitosis [158]. After the metaphase to anaphase transition, concomitantly with postmitotic PCM disassembly, two distanced centrioles finally separate and assemble independent PCM (Figure 4C). By electron microscopy, separated G1 centrioles have usually lost orthogonal orientation, with respect to the mother centriole, and are generally at an increased distance from one another. This final separation step marks the end of centriole engagement.

Plk1 is also thought to participate in the final separation of centrioles in late mitosis [159,160]. Plk1-mediated phosphorylation of Pericentrin in mitosis and its subsequent cleavage by the cysteine protease Separase was shown to be important for the ability of centrioles to separate and duplicate in the subsequent cell cycle [160,161,162]. However, Separase involvement in centriole separation may not be absolutely required or universal. For instance, in human HT116 cells, inactivation of the Separase gene slowed but did not prevent disengagement [159] and in *C. elegans* embryos, depletion of Separase affected centriole disengagement in meiotic but not in mitotic cycles [163]. Further, cleavage of cohesin by Separase was found to be a critical factor for centriole disengagement in U2OS cells [164], but not in HCT116 cells [159] or flies [165]. Thus, more work will need to be done to dissect the events which regulate a timely separation of mother centrioles and procentrioles in mitosis and G1.

#### 4.1.6. Stabilization and Maturation of Disengaged Procentrioles in G1^2^—Centriole-to-Centrosome Conversion

From S^1^ until the end of mitosis^1^, procentrioles do not have the ability to nucleate MTs [166,167] and are not known to perform any function. During that time, procentrioles need to elongate and structure their MT wall. However, they also need to acquire a set of biochemical modifications, which will enable them to accumulate PCM, become stable, and to duplicate once they disengage from mother centrioles. This part of the procentriole maturation process is known as centriole-to-centrosome conversion (CCC) [166]. CCC is not well understood on a molecular level, but removal of several centriolar proteins, which are localized in the procentriole’s lumen or adjacent to their MTs, interferes with conversion, leading to the destabilization and loss of procentrioles after mitosis^1^.

In humans and *Drosophila*, CCC requires molecular interactions between Cep135/Bld10 and Cep295/Ana1, which associate through their N-terminal regions localized adjacent to procentriole MTs (Figure 4) [168,169]. Cep295/Ana1 is thought to bridge the centriole lumen with the centriole periphery and PCM components, which can explain why centriole’s lacking Cep295/Ana1 fail to undergo CCC. Indeed, loss of Cep295 prevents recruitment of Cep192 [170], which in human cells localizes at procentrioles in G2^1^ phase and is important for recruitment of PCM components including duplication factor Cep152 [103,171,172,173]. Similarly, in flies, Ana1 is needed for recruitment of duplication factor Asterless [168,169,174,175]. Cep295/Ana1 incorporates at the proximal ends of procentrioles during S^1^ phase [141,168,170,176]. In human cells, procentriole destabilization due to Cep295 depletion was prevented if procentrioles retained their cartwheel after mitosis^1^ [176]. However, in flies, where centrioles do not physiologically lose their cartwheel after maturation, Ana1-negative centrioles were still lost from the population [168]. Thus, in this system, the cartwheel could not compensate for the loss of CCC. It is important to note, however, that Cep295-negative procentrioles with a cartwheel were not followed past G1^2^ phase, so it is not clear whether they would remain stable during further cell cycle progression.

Cep295/Ana1 depletion additionally results in the formation of short and MT-less procentrioles, while overexpression of Cep295/Ana1 resulted in longer centrioles [141,168]. Further, short procentrioles lacking Cep295 also failed to incorporate more distally positioned inner scaffold proteins POC1B and POC5 [141,177]. Centriolar Cep295 levels can also be perturbed by depletion of PPP1R35 [139], which is enriched in the procentriole proximal lumen above the cartwheel and is required for structuring of procentriole distal ends (Figure 4B) [140]. It is not clear how PPP1R35, which is recruited to procentrioles more distally than Cep295 [140], interferes with Cep295 localization. Finally, depletion of Cep44, a POC1B interacting protein that associates with G2 procentrioles in a Cep295-dependent manner, perturbs centriole MT arrangement and leads to the loss of centrioles [178]. In Cep44 depleted cells, Cep295 was still localized to procentrioles, but was not sufficient for CCC in the absence of procentriole MT integrity. Consistent with this result, centriole destabilization also occurs after co-depletion of POC1A and POC1B [179]. Cep135/Bld10 and POC1 are also required for the assembly and maintenance of triplet MTs of the centriole/basal bodies in *Tetrahymena* and *Paramecium* [78,180,181]. Cep295 is not conserved in nematodes but in *C. elegans*, SAS-7, which localizes proximal to the MT wall, fulfills a similar role in regulating centriole structure and recruitment of the Cep192 homolog Spd-2 [182]. Finally, centrioles are also lost in human cells lacking ε- and δ-tubulin. In these cells, procentrioles form in every cell cycle, but they do not form triplet MTs, fail to incorporate PCM components, and destabilize after mitosis^1^ [183]. Clearly, we are only starting to understand how centriole structural integrity relates to CCC, both of which depend on the concerted action of numerous centrosomal proteins localized near procentriole MTs that bridge the centriole lumen with its periphery.

### 4.2. From Daughter Centrioles to Mother Centrioles and Basal Bodies

From G1^2^ until G1^3^, disengaged and stabilized daughter centrioles continue to mature, which manifests through the loss or recruitment of various proteins (Figure 4). Biochemical and structural asymmetries between the daughter centriole and older mother centriole are reduced by G1^3^, leaving the two centrioles almost indistinguishable from each other.

#### 4.2.1. Acquiring the Ability to Duplicate, Nucleate Microtubules, and Form a Mitotic Spindle Pole

In G1^2^ phase, the daughter centriole can be distinguished from the mother centriole by lower levels of PCM components [167] and, at least in some cell types, by highly motile behavior in comparison to the mother centriole [38]. The centrosome organized by the mother centriole is surrounded by an array of nucleated and anchored MTs, while the daughter centriole has little or no ability to anchor and nucleate MTs [38]. A molecular linker composed of C-NAP1 (CEP250), Rootletin, CNTLN, Cep68, and several other proteins establishes a connection between the two centrosomes [41,184,185,186,187,188] such that the two PCMs organized by each centriole remain adjacent but autonomous. The inter-centrosomal linker is dissolved in G2 phase, allowing the two centrosomes to move away in preparation for mitosis. Mutations and disruptions of the centrosomal linker are associated with genetic disorders and chromosomal instability as reviewed in [184]. For instance, C-NAP1 mutations cause Seckel-like syndrome in cattle [189] and Usher Syndrome in humans [190]. A splice mutation in Rootletin promotes chromosomal instability and chromosome segregation defects in human colorectal cancer cells [191].

The post-mitotic daughter centriole is considered duplication incompetent because it is associated with little or no Plk4. In human cells, Plk4 is recruited through two recruiting factors, Cep192 and Cep152 [100,101,192], which compete for binding to the cryptic polo box domain of Plk4 [103]. While the daughter centriole is associated with Cep192 immediately after mitosis, Cep152 is gradually recruited to the proximal end by its association with Cep63 [28,99]. Cep63, in turn, binds inner PCM protein Cep57, which anchors the Cep63/Cep152 complex to the centriole [27,193]. Per one model [102], Plk4 is initially recruited by Cep192 closer to the centriole, but is repositioned further from the centriole later in G1 as the Cep57/Cep63/Cep152 complex accumulates at the centriole [103,194,195] and Cep152 competes with Cep192 for Plk4 binding.

In addition to duplication factors, throughout G1^2^, S^2^, and G2^2^, the daughter centriole (later the younger mother centriole) continues to accumulate other PCM components such as γ-tubulin, Cep192, Pericentrin, and Cdk5Rap2 (Cep215), which eventually renders it competent for MT nucleation. Finally, before mitosis^2^, PCMs of both the older and the younger mother centriole expand in size in a process called centrosome maturation (Figure 2 and Figure 4) [196,197,198]. This process transiently increases the MT-nucleating capacity of centrosomes, promoting formation of two poles of the mitotic spindle. Centrosome maturation is largely driven by the activity of Plk1 [151], which peaks at the time of prophase [147]. Before mitosis, two mother centrioles accumulate comparable levels of PCM components. This is important for the symmetric organization of the mitotic spindle, accurate segregation of genetic material, and for generating two equal somatic sister cells with similar proliferating potential. Nevertheless, some functional asymmetries between the older and the younger centrosome still persist in mitosis, biasing chromosome segregation [199]. Biochemical and functional asymmetry between two centrosomes is also enhanced and used to generate asymmetric cell divisions during embryonic development, tissue regeneration, morphogenesis, or to influence cell geometry and function [200,201,202,203].

#### 4.2.2. Assembly of Subdistal and Distal Appendages

The assembly of centriole subdistal and distal appendages (SDAs and DAs, respectively), which starts in G2^2^, continues through mitosis and finishes in G1^3^, represents the end of the centriole’s assembly and maturation process (Figure 4). By electron microscopy, SDAs manifest as robust, often striated, usually cone-shaped dense structures emanating from the centriole’s wall ~150–170 nm from the centriole distal end (Figure 1C) [20,36]. The number and shape of SDAs is highly variable [35,36] even between the cells of the same cell type. The ends of SDAs are associated with MTs [39,40], which mediate centrosome positioning, directional cell migration, and centrosome cohesion [37,204,205]. DAs are positioned distally to SDAs [19,20,21,36,79] and look like nine finger-like protrusions, slightly curved toward the centriole distal end [19] (Figure 1A). They have a triangular fibrous base associated with two adjacent MT triplets, a narrower stem, and an electron denser head [19]. DAs are essential for ciliogenesis since they mediate the attachment of ciliary vesicles to mother centrioles and their subsequent fusion with the cytoplasmic membrane (Figure 1C) [31,32,33,34,206,207,208].

Accumulation of SDA and DA components begins on younger mother centrioles in G2^2^ and it involves a sequential incorporation of these components from the centriole’s MT wall toward the periphery [19,31,209,210,211]. The assembly of SDAs is initiated by the recruitment of Odf2 around the centriole’s MTs in G2^2^, followed by the sequential recruitment of SDA components Cep128, CCDC68, CCDC120, Nde1, Centriolin, Ninein, and Cep170 (Figure 4D) [209,211,212]. The assembly of DAs starts with the recruitment of C2CD3, followed by CCDC41/CEP83 in G2^2^. These are inner DA components, localized closer to the centriole wall [19,210]. CCDC41 is needed for the recruitment of CCDC123/Cep89 and SCLT1. The recruitment of SCLT1 begins in late G2^2^ and is, in turn, needed for the recruitment of the outer DA components FBF1 and Cep164 [31] in late mitosis^2^ [19]. Similar timing of the recruitment of DA components to younger mother centrioles has recently been corroborated by [213]. The accumulation of DA components and their organization in a nine-fold symmetrical pattern is continuous throughout mitosis^2^ and for outer DA components even in early G1^3^, as demonstrated by the use of super resolution imaging [19].

In cycling cells, both types of appendages present on mature centrioles in G1, S, and the beginning of G2 undergo a transient remodeling at the end of each cell cycle. SDA electron densities become undetectable in G2, remain undetectable throughout mitosis, and re-appear again in G1. Similarly, DA electron densities become less conspicuous in late G2 and mitosis, and gain their recognizable electron density in the ensuing early G1 [19]. Such diminished detectability of appendages in G2 and mitosis is a consequence of displacement of some appendage components from appendage sites. For instance, in G2, SDA components Cep170 [214] and Ninein [215] relocate from SDAs to the PCM, while Odf2, which is localized adjacent to the centriole, remains associated with centrioles during the entire cell cycle and even increases in intensity in mitosis [19,213]. Similarly, outer DA components Cep164, FBF1, ANKRD26, and TTBK2 dissociate from DAs in G2 and start reaccumulating in late mitosis [19,213,216]. However, DA inner components, CCDC41 and SCLT1, remain permanently associated with mature centrioles [19,213] and are organized in their typical nine-fold pattern [19]. Based on super resolution analyses and the co-localization of CCDC41 and SCLT1 with the DA’s electron dense base, it has been proposed that the inner appendage proteins may serve as a permanent DA scaffold for recruitment of more dynamic, functional outer appendage components [19].

It is not exactly clear why both types of appendages undergo pre-mitotic remodeling. However, it is important to note, that by the end of mitosis, the older and younger mother centrioles still retain certain functional asymmetries. For instance, in RPE-1 cells, the sister cell that inherits the older mother centriole after mitosis tends to grow a primary cilium sooner than its sister harboring the younger mother centriole [217]. So, it has been suggested that pre-mitotic DA remodeling could serve to reduce the age difference between two mother centrioles during mitosis and in early G1 [19]. According to this idea, a transient dismantling of appendages on the older mother centrioles would serve to balance appendage-associated functions between the two sister cells in early G1, before the younger mother centriole’s SDAs and DAs are fully assembled. Indeed, this hypothesis has recently received a direct confirmation [213]. Dissociation of DA components before mitosis was abolished by inhibition of Nek2 kinase activity and perturbed the resorption of primary cilia, which normally occurs by prophase. This in turn, resulted in the asymmetric inheritance of ciliary signaling components and a faster cilium reassembly after cell division [213]. Delayed cilia resorption and retention of Cep164 on appendages throughout mitosis was also observed after Plk1 inhibition [19,213]. This data is compatible with the role of both mitotic kinases in reorganization of mother centriole components before mitosis [218]. It has yet to be understood whether transient remodeling of appendages on the older mother centrioles serves to balance other centriole-associated functions between the two sister cells.

## 5. Regulation of Centriole Maturation by Plk1

How procentriole assembly and maturation are gradually regulated through three cell cycles is not clear. Centriole maturation follows a strict cell cycle-dependent timeline, and kinases, especially Plk1, seem to play important roles in regulating procentriole maturation in both human [166,219] and *Drosophila* cells [220]. Plk1 does not seem to be required for procentriole initiation, which occurs normally in the presence of Plk1 chemical inhibitors [72,155]. However, Plk1 is necessary during G2^1^ phase and mitosis^1^ for some aspects of the centriole maturation process, since its inhibition during that time abrogates centriole-to-centrosome conversion [166]. Furthermore, inhibition of Plk1 during a centriole’s second cell cycle, after CCC has occurred, prevents the assembly of centriole distal appendages, meaning that in cycling cells, Plk1 activity is still needed for maturation of distal ends of daughter centrioles [219].

Conversely, untimely expression of exogenous, active Plk1, which abolishes the physiological periodic activity of Plk1 in cycling cells, accelerates maturation of procentrioles and daughter centrioles, erasing the age difference between resident centrioles [219]. Consequentially, cells acquire supernumerary mature centrioles that form additional primary cilia and increase the overall centriole number due to precipitous distancing of procentrioles and reduplication of mother centrioles. Similarly, in non-cycling, S phase arrested cells, ectopic Plk1 expression induces procentrioles to complete their maturation cycle within one cell cycle, without transition through mitosis. During such an accelerated maturation process, procentrioles undergo typical biochemical and ultrastructural changes associated with physiological procentriole maturation: elongation; acetylation and polyglutamylation of procentriole MTs (as described in the ensuing chapter); association with PCM components; distancing and separation of procentrioles from mother centrioles; licensing for duplication through accumulation of Cep192, Cep63, and Cep152; loss or reduction of procentriole-elongation factors such as Cep120 and Centrobin [221,222]; and assembly of appendages [157,219]. Interestingly, one of the manifestations of procentriole maturation is a robust localization of Plk1 along centriole MT walls, which is barely detectable on immature procentrioles [157]. After its initial recruitment, Plk1 remains associated with mature centrioles. How Plk1 is first recruited to human procentriole MTs is unclear but in flies it requires SAS-4/CPAP [157,220]. Biochemical modifications that occur during centriole maturation appear to be stable, since fully mature centrioles can be maintained after an extended period of Plk1 inactivity [72]. It remains to be understood how exposure to Plk1 modifies centrioles, but one possibility is that Plk1-driven phosphorylation promotes interactions between some centriolar proteins situated on both sides of centriole MTs. Indeed, Plk1 localization along centriole MT walls would perfectly serve this purpose, although at a sufficient concentration, Plk1 could also induce maturation by promoting interactions of centrosomal proteins in the cytosol. It will be fascinating to learn how Plk1 regulates these interactions.

The role of Plk1 in centriole maturation is not limited only to cycling cells. There are specialized, terminally differentiated, multiciliated cells which produce a large number of procentrioles that mature and convert into basal bodies and assemble motile cilia [223,224,225,226]. In multiciliated mouse brain cells, Plk1 is involved in centriole elongation, procentriole distancing, maturation, and migration to the cell surface, which are all critical for multiciliation [227,228].

It is unclear why human procentrioles formed in the absence of Cep295, POC1A/B, PPP1R35, Cep44, or ε- and δ-tubulins fail to undergo CCC even after exposure to Plk1. However, one common theme which emerges from ultrastructural and microscopy analysis of centrioles in cells depleted for these proteins is that they all carry some type of biochemical or centriolar structural defect, even before they were exposed to increased Plk1 activity that would normally result in their maturation and stabilization. Such structural defects may prevent Plk1 from being recruited to procentriole MTs or counteract some Plk1-dependent modification. Per CCC’s broad definition, any novel protein whose depletion or mutation leads to the formation of structurally unstable centrioles could be classified as “required for CCC”. It will be important to start refining this classification by carefully dissecting the hierarchical incorporation of centriolar components in time and space and by meticulously characterizing ultrastructural defects that occur after removal or mutation of each protein.

Finally, the level of centriole maturation is inversely proportional to the ability of centrioles to elongate. Although all centrioles are exposed to the same cytoplasmic pool of centrosomal proteins, only immature centrioles elongate, while mature centrioles maintain their previously established length. This raises an attractive possibility that Plk1 plays a dual role during centriole assembly. Through association with multiple centriole elongation and maturation factors, Plk1 could couple these two opposing processes, establishing some control of the centriole size during unperturbed cell cycles.

## 6. Posttranslational Modifications of Centriole Microtubules—What are They Good for?

**Figure 5:**
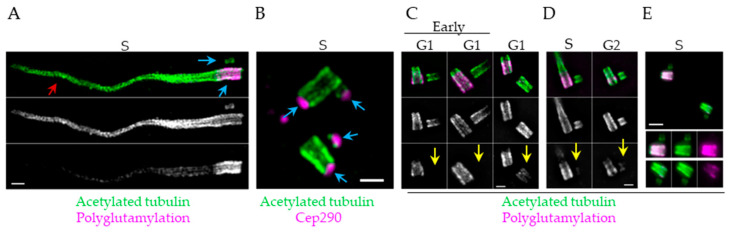
Acetylation and polyglutamylation of centrioles and cilia. Figure illustrates centrioles and basal bodies with cilia from cultured cells. Cells were expanded 4.2-fold (as detailed in Ref. [237]) and imaged by structured illumination microscopy (SIM, **A**–**D**) or conventional widefield microscope using 60× lens (**E**). Centrioles and cilia were co-immunolabeled with antibodies recognizing acetylated tubulin (Sigma; T7451) and centriole ‘cap’ protein Cep290 (Abcam; ab84870) or polyglutamylated tubulin (using an antibody which recognizes a chain of 4 or more glutamates, Adipogen (rabbit, AG-25B-0030-C050)). (**A**) Duplicated older mother centriole from mIMCD3 (mouse inner medullary collecting duct) cell associated with a cilium. Both centrioles (blue arrows) and the ciliary axoneme, (red arrows) are acetylated. Mother centriole and ciliary axoneme are also polyglutamylated. (**B**) Duplicated mother centrioles from HeLa cells associated with short procentrioles. Cep290 ‘caps’ distal ends of centrioles (blue arrows). Note that very short procentrioles are already acetylated. (**C**) G1 centrioles from RPE-1 cells. Mother centrioles are acetylated along the entire MT length but polyglutamylation signal is not present on distal ends. Daughter centrioles still lack or have low levels of polyglutamylation in G1 (yellow arrows). (**D**) Examples of centrioles from mIMCD3 cells. Mother centrioles are acetylated and polyglutamylated. Polyglutamylation signal associated with procentrioles is low (yellow arrows). (**E**) Examples of two duplicated mother centrioles from a HeLa cell in S/G2 phase. One (older) mother centriole is more polyglutamylated than the younger mother centriole. Scale bars: 1000 nm.

During their maturation, procentrioles additionally acquire posttranslational modifications. Centriolar α- and β-tubulins are heavily modified by acetylation, glutamylation, glycylation, and detyrosination [229,230,231]. In addition to centrioles and ciliary axonemes (Figure 5), acetylation is enriched on stabilized cytosolic MTs, with a predominant acetylation site on lysine 40 (K40) of α-tubulin [232]. Acetylation sites other than K40 have been identified on α- and β- tubulins [233,234] which modulate the dynamics of MT nucleation and stability [235,236]. Mature human centrioles are heavily acetylated across the entire MT length (Figure 5). Recent analysis of procentriole acetylation by expansion microscopy showed that procentriole MTs are acetylated from their earliest stages (Figure 5B), with acetylated signal increasing gradually from proximal to distal procentriole ends from S^1^ phase until G1^2^ [237]. By the time the procentrioles finish mitosis^1^, the entire length of MTs is acetylated, although the acetylation signal still shows a proximal–distal gradient until middle G1^2^ (Figure 5C) [237].

Centrioles are also heavily glutamylated (Figure 5). Glutamylation occurs on α- and β-tubulin C-terminal tails that protrude above the MT lattice. Glutamate chains can be of various lengths and they regulate interactions between MT-interacting proteins, as well as the stability and function of microtubules [238,239]. Injection of a monoclonal antibody (GT335) specific to octapeptide EGEGE*EEG, with glutamate on the fifth E residue, compromised centrosome integrity in mitosis [240]. So, glutamylation is widely considered to stabilize centrioles. However, while overexpression of deglutamylase CCP5 removed glutamylation detectable by GT335 antibodies, it did not destabilize centrioles or prevent their PCM recruitment [178]. On mammalian mature centrioles, an antibody recognizing a chain of ≥4 glutamates, shows that the polyglutamylated signal does not occupy the entire centriole length and is more concentrated around the centriole’s proximal ends (Figure 5C). This is consistent with the finding that the polyglutamylation signal is localized at the outer, C tubule of MT triplets [241], which is naturally missing from distal ends. Polyglutamylation signal is, in some cell types, detectable on procentrioles already in S^1^, but it remains of significantly lower intensity throughout the next cell cycle (Figure 5D), reaching the levels of mature centrioles sometimes only in mitosis^2^. In addition, the levels of polyglutamylated signal can be very different between two mother centrioles of the same cell (Figure 5E). This relatively late and unevenly distributed posttranslational modification would be more consistent with some functional role rather than a role in centriole stability. It has yet to be understood how posttranslational modifications of centrioles affect centriole structuring, stability, and function.

## 7. Perspectives

Anomalies in centrioles, centrosomes, and centrosomal proteins have been heavily researched in their connection to cancer and genetic disease [242,243,244,245]. In addition, recent findings identified centriolar structural and numerical defects as promoters of tumorigenesis and tumor invasion and are bringing the centriole and the centrosome to the limelight again. Although most centriolar ultrastructural features have been described by electron microscopy in the seventies and eighties of the last century, with so many components packed in such a small volume and interacting with each other in complex patterns, unraveling the centriole assembly process and the roles of individual centrosomal components is not an easy task. The accessibility of precise genetic tools and super resolution microscopy approaches are accelerating our understanding of centriole ultrastructure and our understanding of the relationship between centriolar structure and function. Many old unanswered questions can now be revisited and reanalyzed with a sophisticated set of tools. Although many concepts and a basic understanding of centriole assembly were established early on, the evolving precision of modern-day imaging and genetic tools promises exciting days ahead for centriole and centrosome researchers.
